# Associations between dialysate interleukin-6 and Tie-2 and peritoneal solute transport rate and outcomes for patients undergoing peritoneal dialysis: A prospective cohort study

**DOI:** 10.12669/pjms.37.4.4328

**Published:** 2021

**Authors:** Ying Hang, Hao Yan, He Zhang, Zhenyuan Li, Wei Fang

**Affiliations:** 1 Ying Hang, Department of Emergency, Renji Hospital, School of Medicine, Shanghai Jiao Tong University, Shanghai, China; 2 Hao Yan, Department of Nephrology, Renji Hospital, School of Medicine, Shanghai Jiao Tong University, Shanghai, China; 3 He Zhang, Department of Nephrology, Renji Hospital, School of Medicine, Shanghai Jiao Tong University, Shanghai, China; 4 Zhenyuan Li, Department of Nephrology, Renji Hospital, School of Medicine, Shanghai Jiao Tong University, Shanghai, China; 5 Wei Fang, Department of Nephrology, Renji Hospital, School of Medicine, Shanghai Jiao Tong University, Shanghai, China

**Keywords:** Angiogenesis, IL-6, Tie-2, High peritoneal solute transport rate, Peritoneal dialysis

## Abstract

**Objectives:**

We designed this prospective observational study to clarify the associations between dialysate IL-6, a marker of ongoing peritoneal inflammation, Tie2, an important factor in angiogenesis in the peritoneum, and a high peritoneal solute transport rate (PSTR) in patients undergoing peritoneal dialysis (PD) and to investigate their outcome predictive roles.

**Methods:**

A total of 60 stable continuous ambulatory peritoneal dialysis (CAPD) patients from a single center in China were analyzed in this prospective study. We measured dialysate levels of IL-6 and Tie-2 using ELISAs. Our primary study endpoint was all-cause mortality with 10 years’ follow-up.

**Results:**

For the evaluation of PSTR, we used the Dialysis/Plasma creatinine (D/Pcr) ratio. We subdivided the patients into two groups for statistical evaluation: low and low average D/Pcr (<0.64; L/A), and high and high average D/Pcr (≥0.65; H/A) transporters. The mean levels of dialysates IL-6 (21.71 ± 8.88 pg/mL) and Tie-2 (1.23 ± 0.43 ng/mL) were significantly higher in the H/A (high and high average, group than those in the L/A group (13.94 ± 5.43 pg/mL, p<0.001 and 0.95 ± 0.43 ng/mL, p=0.019; respectively). Moreover, IL-6 and Tie-2 were positively correlated with D/Pcr (r=0.366, p=0.004 and r=0.402, p=0.001; respectively). Both dialysates IL-6 and Tie-2 were independent determinants of a high peritoneal solute transport rate. After follow-up for 42.65±18.08 months, 30 patients (50.0%) had died. An increased D/Pcr increased the risk of all-cause mortality in patients with CAPD (p=0.018), but the dialysates IL-6 and Tie2 were not independent predictors of all-cause mortality (p>0.05).

**Conclusion:**

Our results suggest that patients undergoing CAPD have a high peritoneal solute transport status with local peritoneal inflammation and angiogenesis. Increased D/Pcr is a relative risk factor for mortality and technique failure in patients undergoing CAPD.

## INTRODUCTION

Peritoneal dialysis (PD) is a well-established renal replacement therapy for patients with end-stage renal disease (ESRD).^1^ Long-term preservation of peritoneal membrane function is a prerequisite for successful PDs. The standard peritoneal equilibration test (PET) is used to estimate the peritoneal small-solute transport rate.^2^ Patients undergoing PD can be classified into high (H), high average (HA), low average (LA), and low (L) PET transporter types. The morphology and function of the peritoneal membrane changes during long-term continuous ambulatory peritoneal dialysis (CAPD) treatment, particularly in patients with high peritoneal solute transport rates (PSTRs) for small solutes.^3^ Angiogenesis, vasculopathy, and sub-mesothelial fibrosis are typical histomorphological alterations of the peritoneal membrane^4^ with predisposing factors including long-term exposure to bio-incompatible dialysis fluid, recurrent peritonitis, and uremia. High concentrations of glucose in the dialysis fluid, glucose degradation products (GDPs), and advanced glycation end-products (AGEs) induce inflammation, fibrosis, and angiogenesis via different pathways.^5^ The peritoneal permeability increases gradually with the duration of the PD treatment, while the peritoneal ultrafiltration function and volume decrease.^6^,^7^ A high peritoneal permeability increases the risk of mortality and technical failure, and ultrafiltration failure has been associated with a high small-solute transport rate, as evidenced by a high D/Pcr.^8^,^9^

Interleukin-6 (IL-6) is a pleiotropic cytokine with roles in multiple pathological and physiological processes and a central regulator of inflammatory processes. Local intraperitoneal IL-6 production is a sign of an intraperitoneal inflammatory state.^10^

Tie-2, a transmembrane tyrosine kinase receptor for Ang ligands crucial for angiogenesis, blood vessel maturation, and vascular endothelium integrity.^11^ Serum Tie2 levels have been associated with different pathologies. Thus, the Ang/Tie-2 system is a primary regulator of angiogenesis and is involved in inflammation.^12^

We hypothesized that intraperitoneal inflammation and angiogenesis are interrelated and associated with a high peritoneal solute transport status in patients undergoing PD. In this study, we analyzed the intraperitoneal IL-6 and Tie-2 levels in 60 patients undergoing stable CAPD treatment with glucose-based solutions. Moreover, we assessed the association between intraperitoneal IL-6 and Tie-2, and their possible correlation to the PSTR during stable CAPDs.

## METHODS

We performed this prospective observational study with data from a single center in China. We recruited all the study participants between July and December of 2007 and prospectively followed them to the end of the study 10 years later (January 31, 2018). All procedures followed in this study were in accordance with the ethical standards of the responsible committee on human experimentation (institutional and national) and with the Helsinki Declaration of 1975. The Human Research Ethics Committee of Renji Hospital, School of Medicine, Shanghai Jiao Tong University approved the study protocol (Approval Number 2016101K). All participants gave their written informed consent.

The 60 patients on CAPD represented all of the clinically stable patients treated in the center and were regularly followed up in the Dialysis Unit of Renji Hospital affiliated to Shanghai Jiao Tong University School of Medicine. This population consisted of 29 men and 31 women with a mean age of 57.9±13.9 years (range, 18–83 years), treated with CAPD for an average of 34.2±29.1 months (3–149.6 months). Table-I lists the ESRD causes for the patients in our study.

**Table-I:** Contrast of general data between L/A and H/A groups (means ± SD or medians with ranges).

Group	L/A group (n=39)	H/A group (n=21)	*p*-value
Age (years)	56.8 ± 14.3	59.8±13.3	0.44
Gender (men/women)	16/23	13/8	0.12
Diabetes Mellitus	17.9%	14.3%	0.66
Peritonitis (episodes/months)	0.0059	0.0051	0.63
PD duration (month)	27.97(3-80)	30.03 (3-149.6)	0.66
4-h drained volume (mL)	2326.15 ± 142.12	2276.67 ± 124.07	0.19
Over-night drained volume (mL)	2249.74 ± 286.83	2036.19 ± 293.44	0.008
Ultrafiltration (mL/24h)	550.00 (-800-1650)	940.00 (-180-2080)	0.02
e GFR (mL/min/1.73 m^2^)	1.74 (0-8.10)	1.72 (0-6.00)	1.00
residual UV (mL/24h)	428.21 (0-1300)	533.33 (0-2000)	0.93
D/P cr	0.51 ± 0.07	0.75 ± 0.11	<0.001
Kt/V t	2.00 ± 0.40	1.94 ± 0.44	0.59
Kt/V p	1.66 ± 0.32	1.58 ± 0.43	0.42
Kt/V r	0.37 (0-1.41)	0.36 (0-1.29)	0.96
Ccr t (L/w/1.73 m^2^)	58.47 ± 19.82	63.07 ± 21.22	0.41
Ccr p (L/w/1.73 m^2^)	40.68 ± 8.02	44.63 ± 10.03	0.10
Ccr r (L/w/1.73 m^2^)	17.79 (0-82.71)	18.44 (0-60.48)	0.91
n PCR (g/kg/day)	0.87 ± 0.20	0.89 ± 0.13	0.79
CRP (mg/L)	14.06 (3.10-32.80)	9.38 (3.12-15.00)	0.12
Glucose (mmol/L)	6.37 ± 1.44	6.43 ± 1.60	0.88
Albumin (mg/L)	38.56 ± 3.45	35.89 ± 3.58	0.006
dialysate IL-6 (pg/mL)	13.94 ± 5.43	21.71 ± 8.88	0.001
dialysate Tie-2 (ng/mL)	0.95 ± 0.43	1.23 ± 0.43	0.019

Values expressed as means ± SD or medians with ranges (min-max). peritoneal dialysis (PD), estimated glomerular filtration rate (e GRF), residual urinary volume (UV), Dialysis/Plasma creatinine (D/Pcr), Dialyzer clearance of urea multiplied by dialysis time and normalized for urea distribution volume (Kt/V), Kt/V total (Kt/V t), Kt/V peritonaeum (Kt/V p), Kt/V residual renal (Kt/V r), Clearance of creatinine (Ccr), Ccr total (Ccr t), Ccr peritonaeum (Ccr p), Ccr Residual renal (Ccr r), normalized protein catabolic rate (n PCR), C-reactive protein (CRP)

On the night before the first dialysis fluid drain for the study, all the patients underwent a dialysis exchange using a 2.5% glucose solution (Dianeal^®^, Baxter, USA) and recorded its exact time.^13^ We collected 10-mL samples of dialysate and immediately stored them at -70°C. We then initiated a standard fast peritoneal equilibrium test (PET).^14^ Dialysate samples for glucose and creatinine were collected at times 0 and 240 min.

For the evaluation of PSTR we used the D/Pcr ratio.^14^ We divided the patients according to the PET classification into high (D/Pcr≥0.81; n=5), high average (D/Pcr 0.65–0.80; n=16), low average (D/Pcr 0.51–0.64; n=22), and low (D/Pcr≤0.50; n=17) transporters. In addition, we subdivided the patients into only two groups for statistical evaluation: low and low average D/Pcr (<0.64; L/A), and high and high average D/Pcr (≥0.65; H/A) transporters.

We determined the concentrations of cytokines in the dialysis effluent after the night dwell using the ELISA technique. All samples were run simultaneously and in duplicate for each mediator to avoid intra- and inter-assay variations. IL-6 and Tie-2 levels were determined using Quantikine^®^ kits (R&D Systems Inc, USA). Both of the assays are considered highly specific for the cytokines, and we observed no significant cross-reactivity. The plates were read using a microplate reader (Bio-Rad, USA).

We followed up all the patients prospectively from the enrollment of the study until death, cessation of PD, transfer to other centers, or the end of the study (January 31, 2018). We evaluated death from all causes as the primary outcome, and limited the survival analysis to the first event.

### Statistical analysis

We expressed results as mean values ± standard deviations (SDs), and used medians and ranges for non-normally distributed parameters. To compare differences between two transport groups, we used unpaired Student *t*-tests. When normal distribution was not present, we applied a non-parametric analysis (Mann–Whitney test). To compare D/Pcr values with their respective cytokine levels, we applied a linear regression analysis. We generated cumulative survival curves using the Kaplan–Meier method. We also applied the Cox proportional hazards model to estimate the relative risks of all-cause mortality for different variables. We performed all statistical tests using the SPSS software version 25.0 for Windows (SPSS, USA).

## RESULTS

The characteristics of the two transport groups are described in Table-I. We found no significant age, gender, dialysis age, or diabetes prevalence differences between the H/A and the L/A groups. We found similar values for serum C-reactive protein (CRP) (p=0.12) and for peritonitis rates (P=0.63) in both groups. The D/Pcr values were 0.51±0.07 in the L/A group and 0.75±0.11 in the H/A group, with the D/Pcr in the H/A group being significantly higher (p<0.001). The dialysis adequacy and residual renal function in the two groups were also similar. However, the overnight drained volume in the H/A group was significantly lower than that in the L/A group (p=0.008). The net ultrafiltration in the H/A group was significantly lower than that in the L/A group (p=0.02). Finally, the serum albumin was significantly lower in the H/A group than in the L/A group (p=0.006).

### Dialysate cytokines and peritoneal transport rate of solutes

**Fig.1:**
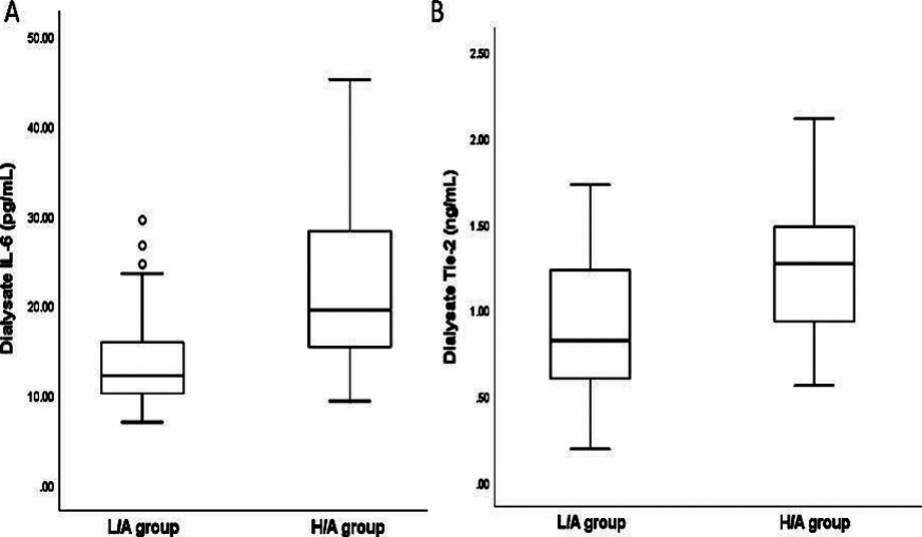
Comparison of dialysate IL-6 (A) and Tie-2 (B) concentrations between L/A and H/A groups Box plot representation: 75% percentile, 25% percentile, median, and maximum and minimum values

**Fig.2:**
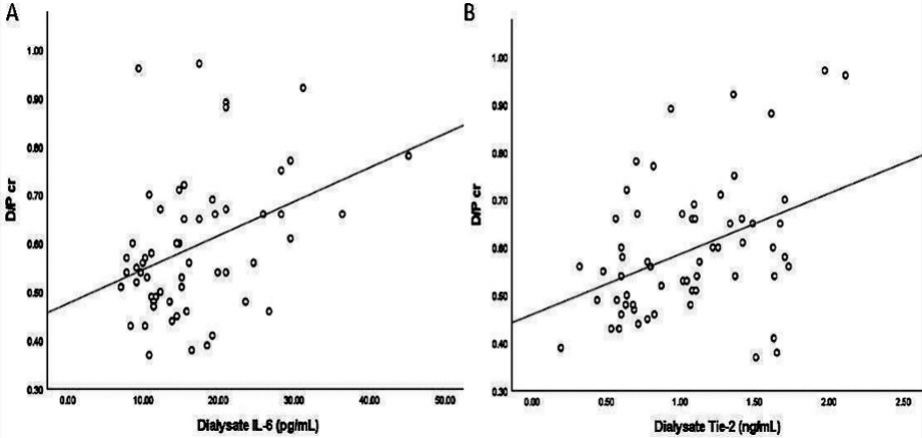
Correlations of dialysate IL-6 (A) and Tie-2 (B) concentrations with D/Pcr levels

The concentration of IL-6 in the dialysate was significantly higher in the H/A group than in the L/A group (p=0.001) (Fig.1A). The dialysate concentration of IL-6 was significantly correlated with the D/Pcr (r=0.366, p=0.004) (Fig.2A).

**Fig.3:**
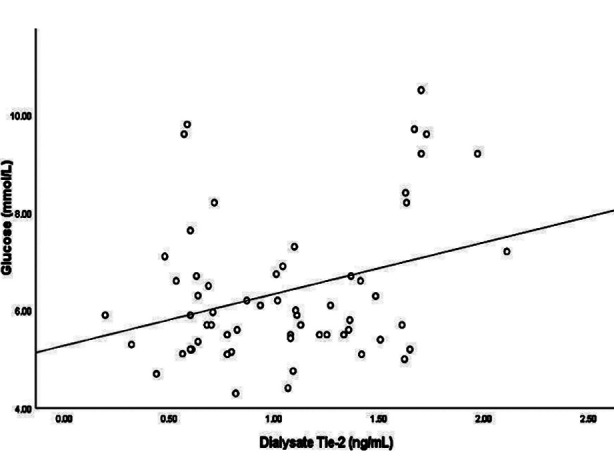
Correlation of dialysate Tie-2 concentration with serum glucose levels

Meanwhile, the concentration of Tie-2 in dialysate was significantly higher in the H/A group than in the L/A group (p=0.019) (Fig.1B). The dialysate concentration of Tie-2 was significantly correlated with both the D/Pcr (r=0.402, p=0.001; Fig.2B) and the blood glucose level (r=0.316, p=0.014; Fig.3). On the other hand, the dialysate concentration of Tie-2 was negatively correlated with the overnight drained volume (r=-0.332, p=0.009). We found no correlation between the dialysate concentrations of IL-6 and Tie-2 (r=0.023, p=0.862).

### Multiple regression analysis of D/Pcr influencing factors

We carried out a multivariate linear regression analysis with age, dialysis age, BMI (body mass index), blood glucose, albumin, CRP, dialysate concentration of IL-6, and the dialysate concentration of Tie-2 as the independent variables, and with the D/Pcr value as the dependent variable. Our results show that the dialysate concentrations of IL-6 (β=0.007, p=0.026) and Tie-2 (β=0.162, p=0.006) were both independent risk factors for D/Pcr (Table-II).

**Table-II:** Multivariate regression analysis for D/Pcr.

D/Pcr		

	Standardized coefficient β	*p*-value
age	0.000	0.478
PD duration	0.000	0.956
BMI	-0.003	0.811
Glu	0.006	0.767
Alb	0.001	0.897
CRP	0.002	0.916
dialysate IL-6	0.007	0.026
dialysate Tie-2	0.162	0.006

### Kaplan-Meier estimates of overall survival probability

**Fig.4:**
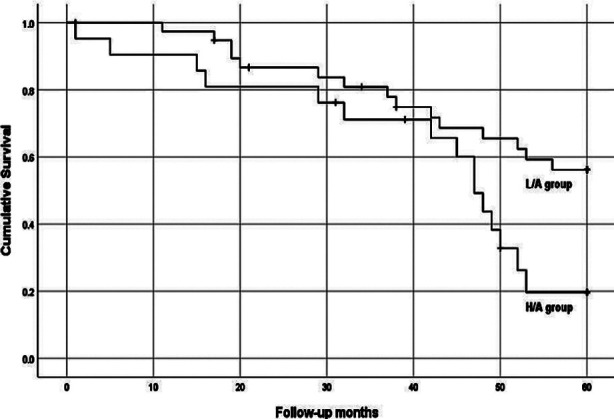
Kaplan-Meier estimates of overall survival probability of patients stratified according to D/Pcr. Low or low average transporters (L/A group; D/Pcr<0.64, 39 patients) and high or high average transporters (H/A group; D/Pcr≥0.65, 21 patients). All-Cause mortality (Log rank=5.629, p=0.018)

After a mean follow-up of 42.65±18.08 months, 30 patients (50.0%) had died and 21 (35.0%) had 21 continued CAPD; two patients (3.3%) had undergone kidney transplantation, 6 (10.0%) had been switched to permanent hemodialysis therapy, and one patient (1.7%) had transferred to another center. According to the Kaplan–Meier survival analysis, the all-cause mortality was significantly higher in the L/A group than in the H/A group (p=0.018; Fig.4), while other factors between the two groups of D/Pcr were similar (all p>0.05).

## DISCUSSION

The patients on PD and with high peritoneal permeability had an increased small-solute PSTR; the peritoneal osmotic pressure gradient disappeared rapidly, resulting in a reduced fluid clearance rate. A high PSTR reflects the large effective peritoneal surface area resulting from a large number of perfused peritoneal capillaries engaged in the diffusion of the solute, and the increased permeability of the peritoneal vasculature. The most common functional alteration during long-term CAPD is an increased peritoneal small-solute transport rate.^15^ Studies have shown that a high peritoneal permeability is an important risk factor for mortality and technical failure in patients undergoing long-term PD.^8^,^9^

We found that patients with higher PSTR had higher levels of dialysate IL-6 and Tie-2, indicating these are closely associated with a small-solute PSTR. Moreover, we found that dialysate concentrations of IL-6 and Tie-2 were independent risk factors of D/Pcr. These results suggest a link between inflammation, angiogenesis, and the small-solute PSTR. However, we found no association between dialysate concentrations of IL-6 and Tie-2.

The acute phase inflammatory response factor IL-6 is secreted by different cells in response to diverse stimuli, it induces the production of acute phase proteins and regulates the temporal switch from acute to chronic inflammation.^16^ Dialysate concentrations of IL-6 reflect an intraperitoneal inflammatory state with cytokine levels being higher in the dialysate than in plasma, showing further increases during peritonitis.^11^ Inflammatory states in patients undergoing CAPD can be due to the use of bio-incompatible dialysis fluids that may induce peritoneal mesothelial cells, macrophages, and endothelial cells to produce of IL-6.^17^ Moreover, high transporter patients undergoing PD have increased levels of dialysate IL-6 that are associated with the PSTR.^13^,^18^,^19^ Longitudinal studies have also correlated the dialysate IL-6 levels with baseline D/Pcrs.^20^ Also, both IL-6 in plasma and dialysate correlate with dialysis adequacy in patients undergoing CAPD.^21^

In this study, the level of dialysate IL-6 was significantly higher in the H/A group than in the L/A group. Moreover, the dialysate concentration of IL-6 had a positive correlation with the D/Pcr. Consistent with the results of a previous study, ours also suggested a close link between a high PSTR and local peritoneal inflammation, and we found that the high peritoneal transport patients presented a more severe local intraperitoneal inflammation state than the others.

IL-6 has been detected in the dialysate of patients lacking signs of systemic inflammation.^22^ In this study, dialysate concentrations of IL-6 did not correlate with the serum CRP concentrations. Local peritoneal production of IL-6 may reflect a chronic inflammatory state of the peritoneum, apart from systemic inflammation. Also, we found a discrepancy in serum albumin levels between the H/A and L/A groups. Hypoalbuminemia has been associated with high peritoneal transport patients undergoing PD.^23^ IL-6 may increase peritoneal leakage of proteins, which may reflect endothelial dysfunction. In addition, significantly prolonged inflammation contributes to a poor nutritional status and higher mortality.^24^ However, hypoalbuminemia in patients undergoing PD is considered a multifactorial condition with dialysis-related and non-dialysis-related factors. Tie-2 can be detected in the blood vessels of normal tissues.^25^

The principal water and solute barriers in PD are the blood capillary endothelium and the interstitial matrix. The peritoneal permeability increases in patients undergoing PD and, eventually, leads to ultrafiltration failure. Peritoneal biopsies in patients undergoing PD have shown that extensive angiogenesis is associated with peritoneal treatment duration.^26^ Angiogenesis is thought to result in an increased effective surface exchange area, whereas lymphangiogenesis leads to raised lymphatic absorption rates. The expanded vascular network results in a decrease in the glucose-driven osmotic pressure of the PD fluid leading to ultrafiltration loss. In addition, vascular wall thickening and increased permeability cause an increase in small solute transport and result in a reduction time for exchanging waste products.^27^,^28^ A cartilage oligomeric matrix protein (COMP)-Ang-1 treatment may significantly increase the level of phosphorylation of the Tie-2 receptor in uremic peritoneal dialysis rats, suggesting that preservation of the Ang-1/Tie-2 signal may alleviate peritoneal membrane injuries and improve the peritoneal transport function.^29^ Peritoneal angiogenesis may be the main factor increasing the peritoneal solute transport.^30^ Markers, such as Tie-2, could be used to obtain information on “peritoneal angiogenesis”. We showed that the dialysate Tie-2 level in the H/A group was significantly higher than that in the L/A group, suggesting that a high PSTR may be due to increased peritoneal angiogenesis. Moreover, we also found that the dialysate Tie-2 levels were positively correlated with the D/Pcr ratio. The dialysate concentration of Tie-2 was an independent risk factor for a high D/Pcr, suggesting that patients undergoing PD who are high small solute peritoneal transporters may present increased peritoneal angiogenesis; we found angiogenesis to be an independent risk factor for peritoneal permeability.

Interestingly, we also found that the dialysate Tie-2 level was positively correlated with the blood glucose level. Glucose is a proinflammatory agent with additional profibrotic effects. A high concentration of glucose in the dialysate may promote angiogenesis and fibrosis of the peritoneal membrane through a stimulation of the polyol pathway.^31^ GDPs produce more AGE products than glucose. GDPs and AGEs are pro-inflammatory factors that can be induced by VEGF-mediated angiogenesis.^4^ We confirmed results from previous studies showing that in addition to a high glucose concentration in the dialysis fluid, the peripheral blood glucose concentration may also promote peritoneal angiogenesis.

Studies have demonstrated an intimate connection between a high transport status and poor outcomes.^8^,^32^,^33^ Meta-analyses have indicated that the relative risks for mortality and technique failure increase by 1.15 and 1.18 (respectively) for every 0.1 increase in the dialysate over plasma ratio for D/Pcr.^7^ The mortalities of the LA, HA, and high transport groups increased by 21.9%, 45.7% and 77.3%, respectively, as compared to that of the low transport group. We found a significant increase in all-cause mortality between the L/A group and the H/A group with a 60-month follow-up confirming that an increased D/Pcr is a relative risk factor for mortality and technique failure in patients undergoing CAPD. Cardiovascular disease (CVD) events were the leading cause of death in our patients, followed by cerebrovascular events. Studies have reported positive associations between circulating Tie-2 and cardiovascular risk factors.^34^,^35^ Meanwhile, chronic inflammation is a remarkable risk factor for atherosclerotic disease particularly in patients undergoing CAPD.^36^ Our results show that the patients in the H/A group had higher levels of dialysate IL-6 and Tie-2 than patients in the L/A group, but we did not test circulating IL-6 and Tie-2 levels in these patients. Therefore, we cannot confirm that dialysate IL-6 and Tie-2 are relative risk factors for mortality and technique failure in patients undergoing CAPD. We can only speculate the existence of an association between intraperitoneal inflammation, angiogenesis, and the outcome of patients undergoing CAPD.

### Limitations

First of all, we only performed single-time-point measurements of dialysate concentrations of IL-6 and Tie-2 that may not reflect changes over time or time-averaged exposures. Second, the number of cases included in this study is relatively small. another well-designed and extensive study will be required to confirm our findings.

## CONCLUSION

In summary, our results suggest that patients undergoing CAPD have a high PSTR with local peritoneal inflammation and angiogenesis. The dialysate concentrations of IL-6 and Tie-2 both seem to be independent risk factors for peritoneal permeability. In our patients, peritoneal local inflammation and angiogenesis were both involved in the peritoneal permeability changes observed. Increased D/Pcr was a relative risk for mortality and technique failure in these patients.

### Authors’ contributions:

**YH and WF** conceived, designed the study and are responsible for integrity of the study.

**HZ and ZL** collected the data.

**HY** performed the analysis.

**YH** was involved in the writing of the manuscript.

All authors have read and approved the final manuscript.
